# Extracellular volume quantification in isolated hypertension - changes at the detectable limits?

**DOI:** 10.1186/s12968-015-0176-3

**Published:** 2015-08-12

**Authors:** Thomas A. Treibel, Filip Zemrak, Daniel M. Sado, Sanjay M. Banypersad, Steven K. White, Viviana Maestrini, Andrea Barison, Vimal Patel, Anna S. Herrey, Ceri Davies, Mark J. Caulfield, Steffen E. Petersen, James C. Moon

**Affiliations:** Department of Cardiology, The Heart Hospital, University College London Hospitals NHS Trust, London, UK; National Institute for Health Research Cardiovascular Biomedical Research Unit at Barts, William Harvey Research Institute, Barts and the London School of Medicine and Dentistry, Queen Mary University of London, London, UK; The Hatter Cardiovascular Institute, University College London Hospitals NHS Trust, London, UK; Fondazione Toscana Gabriele Monasterio and Scuola Superiore Sant’Anna, Pisa, Italy; The Heart Hospital Imaging Centre, University College London Hospitals NHS Trust, 16-18 Westmoreland Street, London, W1G 8PH UK

**Keywords:** Hypertension, Left ventricular hypertrophy, Magnetic resonance imaging, T1 mapping, Myocardial fibrosis

## Abstract

**Background:**

Diffuse myocardial fibrosis (DMF) is important in cardiovascular disease, however until recently could only be assessed by invasive biopsy. We hypothesised that DMF measured by T1 mapping is elevated in isolated systemic hypertension.

**Methods:**

In a study of well-controlled hypertensive patients from a specialist tertiary centre, 46 hypertensive patients (median age 56, range 21 to 78, 52 % male) and 50 healthy volunteers (median age 45, range 28 to 69, 52 % male) underwent clinical CMR at 1.5 T with T1 mapping (ShMOLLI) using the equilibrium contrast technique for extracellular volume (ECV) quantification. Patients underwent 24-hours Automated Blood Pressure Monitoring (ABPM), echocardiographic assessment of diastolic function, aortic stiffness assessment and measurement of NT-pro-BNP and collagen biomarkers.

**Results:**

Late gadolinium enhancement (LGE) revealed significant unexpected underlying pathology in 6 out of 46 patients (13 %; myocardial infarction n = 3; hypertrophic cardiomyopathy (HCM) n = 3); these were subsequently excluded. Limited, non-ischaemic LGE patterns were seen in 11 out of the remaining 40 (28 %) patients. Hypertensives on therapy (mean 2.2 agents) had a mean ABPM of 152/88 mmHg, but only 35 % (14/40) had left ventricular hypertrophy (LVH; LV mass male > 90 g/m^2^; female > 78 g/m^2^). Native myocardial T1 was similar in hypertensives and controls (955 ± 30 ms versus 965 ± 38 ms, *p = 0.16*). The difference in ECV did not reach significance (0.26 ± 0.02 versus 0.27 ± 0.03, *p = 0.06*). In the subset with LVH, the ECV was significantly higher (0.28 ± 0.03 versus 0.26 ± 0.02, *p < 0.001*).

**Conclusion:**

In well-controlled hypertensive patients, conventional CMR discovered significant underlying diseases (chronic infarction, HCM) not detected by echocardiography previously or even during this study. T1 mapping revealed increased diffuse myocardial fibrosis, but the increases were small and only occurred with LVH.

## Background

Arterial hypertension is one of the most common cardiovascular diseases and a major cause of morbidity and mortality in the developed world. Arterial hypertension results in increasing arterial stiffness and afterload, leading to remodelling of the myocardium due to cardiomyocyte hypertrophy, fibroblast stimulation and then increased collagen formation. Progressive accumulation of interstitial collagen fibres, i.e. diffuse myocardial fibrosis (DMF), in left ventricular hypertrophy (LVH) has been shown at necropsy [1] and endomyocardial biopsy [2–4]. The cardiovascular magnetic resonance (CMR) derived late gadolinium enhancement (LGE) technique has shown patchy, non-specific or non-ischaemic patterns of fibrosis in hypertension [5]. However, LGE is only able to detect relative increases between “normal” myocardium and focal scar and cannot therefore be used to identify and quantify absolute diffuse fibrosis [6–8]. This problem can be now addressed by quantification of T1 relaxation time mapping before and after gadolinium contrast administration and subsequent quantification of extracellular volume (ECV). Both ECV and native myocardial T1 have been shown to closely reflect the degree of histologic DMF [9–12]. Preliminary work on DMF in hypertension have described elevated extracellular volume fraction (ECV) compared to controls [13, 14]. However, this technique has not been used to comprehensively assess a cohort of hypertensive patients. We hypothesize that diffuse myocardial fibrosis measured by T1 mapping and ECV quantification is elevated in isolated systemic hypertension, correlates with cardiac remodelling and established biomarkers, and may therefore be a key biomarker in assessing the cardiac effects of systemic hypertension.

## Methods

All research was carried out at University College London Hospital NHS Trust in collaboration with William Harvey Research Institute at Queen Mary University of London between April 2011 and February 2012. The study was approved by the ethical committee of UK National Research Ethics Service and conformed to the principles of the Helsinki Declaration (UK NRES 07/H0715/101). All subjects gave written consent to participate in the study.

**Hypertensive subjects** were recruited prospectively from a specialist hypertension clinic in a tertiary referral hospital. All patients had been investigated for secondary hypertension as part of their clinical work-up in the specialist hypertension clinic. Eligible patients were men and women between 18 and 80 years of age with essential hypertension. In accordance with the 2011 UK hypertension guidelines [15], ambulatory blood pressure measurement (ABPM) was used to confirm diagnosis of recruited patients (clinic blood pressure of ≥140/90 mmHg and daytime ABPM of ≥135/85 mmHg) and patients with “white coat” hypertension (not on anti-hypertensive medications with a normal ABPM) were excluded. Comprehensive assessment on the day of the CMR consisted of clinical history, arterial stiffness and blood pressure measurement following a period of rest, transthoracic echocardiography, electrocardiogram (ECG), blood tests (NT-pro-BNP, full blood count for the haematocrit, renal function, and lipid profile), 6-minute walk test (6MWT), and CMR (including equilibrium diffuse myocardial fibrosis protocol). ECG was analysed for LVH by Cornell product and Sokolow-Lyon voltage criteria [16, 17].

**A control group** of healthy, normotensive volunteers recruited from hospital, university, community and general practice settings in Greater London, UK, were gender matched to the hypertension cohort. None were referred as patients for a clinical CMR scan that then turned out to be normal. All normal subjects had no history or symptoms of cardiovascular disease or diabetes. All subjects had a normal blood pressure (defined as ≤140/90 mmHg), 12-lead ECG and clinical CMR scan.

**Exclusion criteria** for both groups included diabetes mellitus, known ischaemic heart disease, contraindication to CMR (pacemakers) or gadolinium administration (glomerular filtration rate <30 mL/min/m^2^). Healthy volunteers were excluded if they had a history of cardiovascular symptoms, an abnormal ECG or abnormal CMR.

### CMR protocol

Standard CMR examinations were performed in all patients using a 1.5-T scanner (Avanto; Siemens Medical Imaging, Erlangen, Germany) in line with standard CMR protocols [18]. T1 mapping for CMR ECV quantification was performed using the Shortened Modified Look-Locker Inversion recovery technique (ShMOLLI) (21) prior to contrast and at contrast equilibrium [9]. The studies were performed by cardiologists with ≥2 years of experience in CMR imaging (DS, SB, SW). Standard LGE assessment using a fast low angle single shot inversion recovery sequence was used to assess focal myocardial fibrosis. Fifteen minutes after an initial contrast bolus of 0.1 mmol/kg of gadoterate meglumine (Dotarem; Guerbet, Paris, France) during which LGE images were acquired, an infusion of contrast at a rate of 0.0011 mmol/kg per minute was administered during which time the patient was removed from the scanner. After a minimum of 30 minutes the patient was returned into the scanner and equilibrium contrast T1 maps were acquired. The ECV was calculated by: ECV = (1–haematocrit) x (1/ΔT1_tissue_)/(1/ΔT1_blood_). Haematocrit was measured on the same day.

### CMR image analysis

**Fig. 1:**
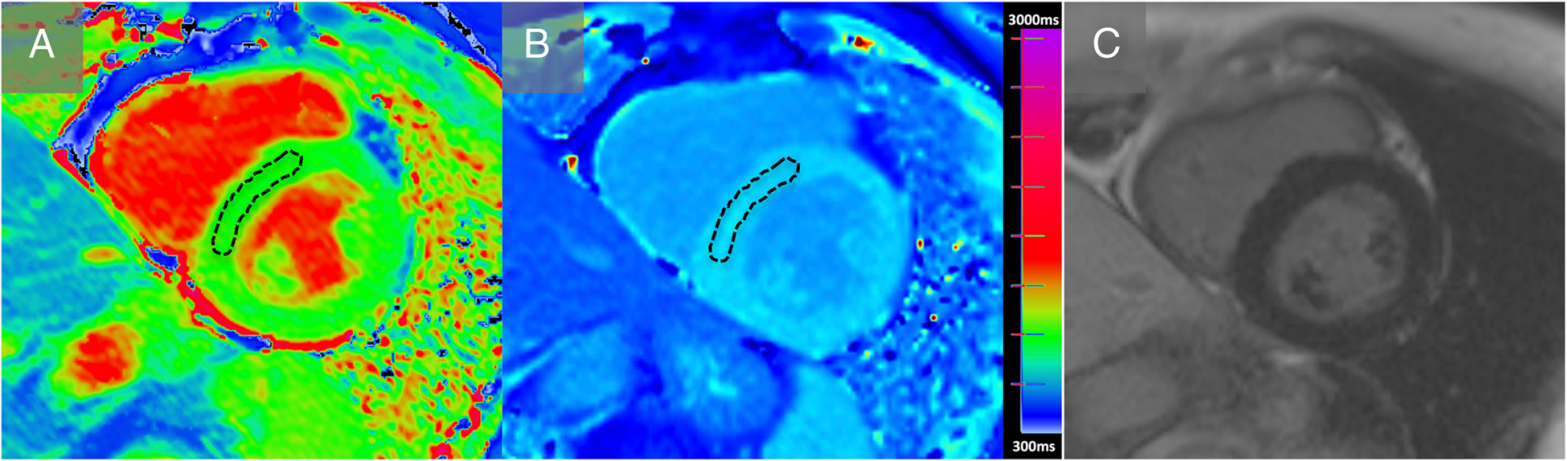
Native and post contrast T1 mapping and LGE imaging in hypertension. Basal short axis T1 maps acquired pre-contrast (**a**) and post contrast (**b**) with ShMOLLI. Image **c** shows the corresponding FLASH LGE image. The dashed black line represents an example region of interest.

Left ventricular (LV) volumes, ejection fraction and mass were calculated using standard techniques [18] and analysed using a thresholding method indexed to body surface area. The presence of LGE was determined using the visual assessment of two authors (TT and JCM, the latter of whom has >10 years of CMR experience). For T1 measurements, a region of interest (ROI) was manually drawn on the septum on each image (TT) as shown in Fig. 1. Our group has previously validated this method (23). Myocardial feature tracking was performed by a blinded experienced observer using dedicated vector-based analysis tool (2D performance analysis MR, Tomtec, Unterschleissheim, Germany), as previously described [19]. Briefly, based on a contour manually drawn by an expert reader along the LV endocardial border of one frame, the software automatically propagates the contour and follows its features throughout the remainder of the cardiac cycle. LV short axis circumferential and radial strains were calculated from a mid-ventricular short-axis view containing both papillary muscles. Strain values (% change from baseline) were obtained for each segment and global values defined as the mean of all segmental values.

### Echocardiography

Echocardiography was performed on the day of the CMR in all hypertensive subjects using a GE Vivid E9 system (GE Healthcare, Wauwatosa, USA) with a 4-MHz transducer. Measurements were made according to European Society of Echocardiography criteria [20]. Early (E) and atrial (A) doppler mitral inflow wave velocities, pulmonary vein doppler, and tissue doppler of septal and lateral mitral annulus E’ and A’-wave velocities were recorded. Diastolic function was graded according to the mitral inflow pattern, pulmonary vein in-flow and tissue doppler indices at the mitral valve annulus as previously described [21].

### Arterial stiffness

Arterial stiffness was measured in hypertensive subjects using the Vicorder device (Skidmore Medical Ltd, UK), by registering carotid femoral pulse wave velocity (PWV), central aortic blood pressure and augmentation index as previously described and validated. (23, 24) PWV is the most validated method to non-invasively measure arterial stiffness. It is considered the gold standard assessment of aortic stiffness, as it is a relatively simple method with reported accuracy, reproducibility and is also an independent and strong predictor of adverse outcomes in a variety of common diseases, such as coronary heart disease and hypertension [22, 23]. The measurements were acquired in a separate quiet room after a period of rest. Central blood pressure measurement and aortic stiffness estimated by pulse wave velocity (PWV) measurement are more predictive of cardiovascular outcome than peripheral BP [23, 24].

#### Biomarkers

Serum levels of the N-terminal pro-hormone of pro-brain-natriuretic-peptide (NT-pro-BNP) were measured using a 2-site electrochemiluminescence immunoassay on a Roche E170 analyzer. Collagen Type I (procollagen type I carboxy-terminal propeptide, (PICP)) and Type III (Amino terminal peptide of type III procollagen ((PIIINP)) synthesis were measured using commercially available assays. The samples were collected at the time of CMR visit, immediately centrifuged and plasma samples were stored at −80 °C.

### Data analysis and statistics

Statistical analysis was performed in R programming language for statistical computing (version 3.0.1, The R Foundation for Statistical Computing) and SPSS version 21 (SPSS Inc, Chicago, Ill). Normality of continuous data was assessed by visual inspection of the histograms and confirmed by the Shapiro-Wilk test. Continuous variables were expressed as mean ± SD and non-parametric variables as median with inter-quartile range. Group mean comparisons were performed using Student’s t-test (two groups) for normally distributed data or the Mann-Whittney U test for skewed data. A probability value of p < 0.05 was considered statistically significant. Simple and multivariable linear regression models determined relationships between LV mass index, native myocardial T1 time, equilibrium myocardial T1 time and ECV as outcome variables, using demographic data, laboratory results and CMR measurements as exposure variables.

## Results

**Fig. 2:**
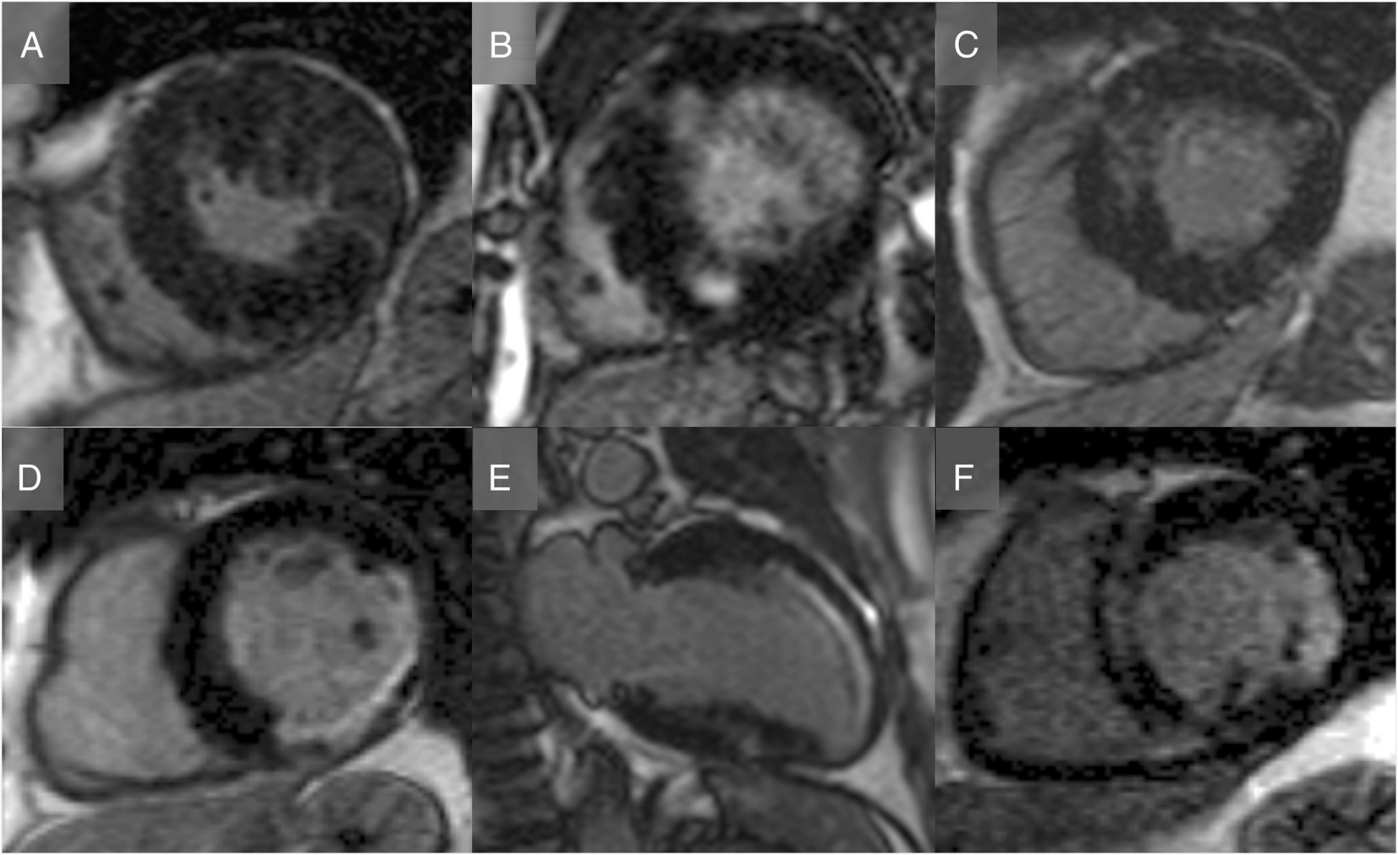
Diagnosis of occult disease by LGE in patients with arterial hypertension. Standard late gadolinium enhancement assessment using a fast low angle single shot inversion recovery sequence revealed six patients (13 % of cohort) with either hypertrophic cardiomyopathy (**a**-**c**) or infarct pattern (**d**-**f**).

Fifty-six well-controlled hypertensive patients were recruited. Ten patients were subsequently excluded as they were found to have white coat hypertension. Forty-six patients and 50 healthy volunteers underwent the full CMR protocol. Morphological, functional and LGE assessment revealed unexpected significant underlying pathology in 6 out of the 46 patients (13 %; chronic infarct (n = 3); hypertrophic cardiomyopathy (HCM, n = 3); Fig. 2). These six patients were excluded from subsequent analysis. The remaining cohort comprised of 40 patients (median age 56, range 21 to 78, 52 % male) and 50 healthy volunteers (median age 45, range 28 to 69, 52 % male). Patient ethnicity was self-reported as Caucasian/white in 72.5 %, Asian in 12.5 % and Afro-Caribbean/black in 15 %. Fifteen (37.5 %) of the study subjects were treated with angiotensin converting enzyme inhibitors, 11 (27.5 %) with angiotensin II antagonists, 25 (62.5 %) with calcium channel blockers, 11 (27.5 %) with thiazide diuretic, 10 (25 %) with beta-blockers, 10 (25 %) with alpha-blockers 4 (10 %) with loop diuretic, 2 (5 %) with moxonidine and 1 (2.5 %) each with spironolactone, direct renin antagonist and phenoxybenzamine. Seven patients were not on any anti-hypertensive therapy. All clinical parameters are summarized in Table 1.

**Table 1:** Baseline clinical characteristics for healthy volunteers and hypertensives

	Healthy volunteers	Hypertensive	
	(n = 50)	(n = 40)	*p value*
Men	26 (52 %)	21 (53 %)	*0.96*
Age in years (IQR)	44 (32.0 to 54.8)	58.5 (49.0 to 65.5)	*<0.001*
Ethnicity			
Whites	41 (82 %)	28 (70 %)	*0.12*
Blacks	8 (16 %)	6 (15 %)	*0.59*
Asians	1 (2 %)	6 (15 %)	*0.06*
Height (cm)	171.9 ± 11.3	170.2 ± 9.1	*0.41*
Weight (kg)	75.9 ± 13.8	86.6 ± 15.7	*<0.01*
Body surface area (m2)	1.9 ± 0.2	2.0 ± 0.2	*<0.05*
Body mass index (kg/m2)	25.6 ± 3.2	29.8 ± 4.5	*<0.0001*
Systolic blood pressure (mmHg)	122.7 ± 10.5	152.0 ± 17.2	*<0.0001*
Diastolic blood pressure (mmHg)	74.1 ± 9.1	88.1 ± 10.7	*<0.0001*
eGFR (ml/min/1.73 m2)	91.2 ± 16.5	81.1 ± 20.8	*<0.05*
Haematocrit (%)	0.42 ± 0.03	0.42 ± 0.04	*0.37*
Number of antihypertensives (0/1/2/3/4/5/6)		7/5/13/7/4/3/1	

### Left ventricular remodelling

The conventional CMR parameters are shown in Table 2. Hypertensive subjects had higher LV mass index (83.9 ± 33.6 vs. 65.7 ± 14.5 g/m^2^, p < 0.01), maximal wall thickness, LV mass to volume ratio (MVR), left atrial area (LAA) measured in the horizontal long axis view, indexed stroke volume (SV), but no significant differences in ejection fraction (EF) or indexed end-diastolic (EDV) and end-systolic (ESV) volumes.

**Table 2:** Cardiovascular Magnetic Resonance Parameters

	Healthy volunteers	Hypertensive	
	(n = 50)	(n = 40)	*p value*
EDV index (ml/m2)	73.2 ± 13.6	71.2 ± 17.0	*0.7*
ESV index (ml/m2)	25.0 (IQR: 21.0 to 29.75)	21.8 (IQR: 15.3 to 28.1)	*0.13*
LVEF (%)	66.5 ± 5.8	69.0 ± 8.9	*0.12*
LAA index (cm/m2)	11.1 ± 1.8	11.7 ± 2.5	*0.16*
Maximal wall thickness (mm)	6.7 ± 1.2	11.9 ± 2.4	*<0.001*
LV mass index (g/m2)	65.0 (IQR: 55.3 to 74.5)	76.9 (IQR: 66.5 to 88.4)	*<0.001*
LV mass/EDV (g/ml)	0.91 (IQR: 0.77 to 1.04)	1.09 (IQR: 0.93 to 1.31)	*<0.001*
LV hypertrophy	0	14 (35 %)	

LVH (defined as LV mass index >90 g/m^2^ in males and >78 g/m^2^ in females [25]) was found in 35 % of patients (14/40). Those who fulfilled LVH criteria had higher maximal wall thickness, but also larger ventricles (as expressed by ESV and EDV) and larger indexed LAA (Table 3). Furthermore, this subgroup had higher central blood pressure, higher QRS complex voltage on ECG (by Sokolov and Cornell) and more advanced diastolic dysfunction (p < 0.01) (Table 3).

**Table 3:** Hypertensives without versus Hypertensives with LVH

	Hypertensives no LVH	Hypertensives with LVH	
	n = 26	n = 14	*p-value*
T1 mapping			
T1 Blood (ms)	1564 ± 61	1614 ± 83	*0.02*
T1 Myocardium (ms)	948 ± 31	997 ± 27	*<0.001*
ECV by ShMOLLI (%)	26.2 ± 2.2	28.8 ± 2.8	*<0.01*
Clinical			
Men	16 (62 %)	5 (35 %)	*0.12*
Age in years (IQR)	57.8 ± 12.2	53.2 ± 17.5	*0.4*
Height (cm)	171 ± 10.4	168.5 ± 6.1	*0.44*
Weight (kg)	86.6 ± 16.9	86.6 ± 13.8	*1*
Body surface area (m^2^)	2.02 ± 0.24	2.01 ± 0.18	*0.79*
Body mass index (kg/m^2^)	29.5 ± 4.7	30.4 ± 4.2	*0.49*
Systolic blood pressure (mmHg)	150.1 ± 18.0	155.5 ± 15.7	*0.35*
Diastolic blood pressure (mmHg)	87.1 ± 11.7	90.0 ± 8.4	*0.35*
eGFR (ml/min/1.73 m^2^)	83.6 ± 18.8	76.4 ± 24.2	*0.23*
Haematocrit (%)	0.43 ± 0.03	0.42 ± 0.05	*0.39*
Number of antihypertensives (0/1/2/3/4/5/6)	3/5/8/6/2/1/1	4/0/5/1/2/2/0	
CMR			
EDV index (ml/m^2^)	66.2 ± 11.7	82.5 ± 20.5	*0.02*
ESV index (ml/m^2^)	19.9 ± 7.2	29.4 ± 14.4	*0.05*
LVEF (%)	70.7 ± 7.3	65.9 ± 10.9	*0.16*
LAA index (cm/m^2^)	10.9 ± 2.1	13.3 ± 2.5	*<0.01*
Maximal wall thickness (mm)	10.9 ± 1.6	13.8 ± 2.6	*<0.001*
LV mass index (g/m^2^)	68.6 ± 11.8	112.4 ± 39.6	*<0.001*
LV mss/EDV (g/ml)	1.07 ± 0.26	1.41 ± 0.5	*0.05*
Transverse strain	34.0 ± 11.8	30.8 ± 9.4	*0.21*
Longitudinal strain	−18.7 ± 5.4	−17.4 ± 3.8	*0.41*
Circumferential strain	−24.7 ± 5.3	−24.6 ± 3.8	*0.64*
Radial strain	37.3 ± 13.1	35.4 ± 7.1	*0.94*
Late enhancement, n (%)	5 (19)	6 (43)	*0.15*
Electrocardiogram			
Sokolov-Lyon index	22.3 ± 4.8	32.5 ± 13.1	*<0.01*
Cornell voltage criteria	13.7 ± 5.2	21.1 ± 8.7	*<0.01*
Aortic stiffness			
Pulse wave velocity	7.7 ± 1.5	8.4 ± 1.6	*0.15*
Augmentation index	17.0 ± 5.7	13.6 ± 6.6	*0.18*
Aortic systolic blood pressure (mmHg)	141.5 ± 14.9	167.4 ± 27.2	*<0.01*
Aortic pulse pressure (mmHg)	54.6 ± 13.4	64.3 ± 17.2	*0.13*
Diastolic function on echocardiogram			
E/A	0.9 ± 0.3	1.1 ± 0.5	*0.7*
E/e’ septal	10.3 ± 2.9	12.9 ± 7.3	*0.36*
E/e’ lateral	8.0 ± 2.3	12.9 8 ± 6.4	*<0.01*
Diastolic grade (I/II/III/IV)	3/18/5/0	0/6/7/1	*<0.01*

Higher LV mass was associated with larger LV volumes and LA size (Table 4): 10.3 g for each 10 ml increase in ESV (*p < 0.01*), 7.4 g for each 10 ml increase in EDV (*p < 0.0001*) and 5.4 g for each 1 cm^2^ increase in LAA (*p < 0.0001*). The severity of diastolic dysfunction (trans-mitral E/A ratio, tissue Doppler lateral E/E’ ratio) and higher ECG voltage parameters (by Cornell and Sokolov) also increased with higher LV mass (data not presented). There were no differences between the groups in the systolic strain parameters derived from feature tracking analysis, in particular in global longitudinal, circumferential or radial strain.

**Table 4:** Univariate Predictors of T1 mapping Parameters and Left Ventricular Hypertrophy

	Native myocardial T1		Equilibrium myocardial T1		ECV		LV mass index	
	Univariable		Univariable		Univariable		Univariable	
	beta	*p*	beta	*p*	beta	*p*	beta	*p*
Men	−9.2	*0.2*	2.49	*0.77*	−1.27	*<0.05*	18	*<0.001*
Age per year	−0.22	*0.32*	−0.09	*<0.001*	0	*0.18*	−0.15	*0.39*
Ethnicity	9.45	*0.11*						
*Asian*			−3.5	*0.76*	0.46	*0.5*	0.97	*0.89*
*Black/Afrocaribbean*			−37.5	*<0.05*	4.07	*<0.0001*	37.45	*<0.001*
Height (cm)	−0.13	*0.72*	0.61	*0.14*	−0.05	*<0.05*	0.46	*0.08*
Weight (kg)	0.11	*0.64*	−0.82	*<0.01*	−0.03	*0.14*	0.51	*<0.01*
Body surface area (m^2^)	4.13	*0.8*	−39.5	*<0.05*	−2.0	*0.09*	35.0	*<0.01*
Body mass index (kg/m^2^)	0.74	*0.37*	−4.75	*<0.0001*	−0.02	*0.72*	1.48	*<0.05*
Systolic blood pressure (mmHg)	0.13	*0.5*	−0.76	*<0.001*	0.01	*0.33*	0.37	*<0.05*
Diastolic blood pressure (mmHg)	0.07	*0.81*	−1.15	*<0.01*	0.01	*0.62*	0.60	*<0.05*
eGFR (ml/min/1.73 m^2^)	0.09	*0.66*	0.88	*<0.0001*	−0.03	*0.09*	−0.42	*<0.01*
Hematocrit (%)	−0.94	*0.42*	0.38	*0.8*	−0.23	*<0.01*	179.7	*<0.05*
EDV index (ml/m^2^)	0.35	*0.15*	0.08	*0.77*	0.03	*0.9*	0.74	*<0.0001*
ESV index (ml/m^2^)	0.23	*0.55*	0.13	*0.77*	0.01	*0.67*	1.03	*<0.001*
LVEF (%)	0.09	*0.85*	−0.18	*0.76*	0.04	*0.32*	−0.68	*0.06*
LAA index (cm/m^2^)	3.12	*0.06*	−0.59	*0.77*	0.34	*<0.01*	5.14	*<0.0001*
Maximal wall thickness (mm)	2.89	*<0.01*	−5.02	*<0.0001*	0.18	*<0.05*	--	--
LV mass index (g/m^2^)	0.40	*<0.01*	−0.33	*<0.05*	0.02	*<0.05*	--	--
LVH (presence)	44.26	*<0.0001*	−28.8	*<0.05*	2.65	*<0.001*	--	--
E/A ratio	−0.15	0.99	16.2	0.3	-0.001	*0.9*	40.2	*<0.01*
E/e’ septum	1.81	0.14	−1.51	0.2	0.002	*0.08*	3.18	*<0.01*
E/e’ lateral wall	3.21	*<0.01*	−1.23	0.3	0.001	*0.3*	3.76	*<0.001*

### Late gadolinium enhancement

**Fig. 3:**
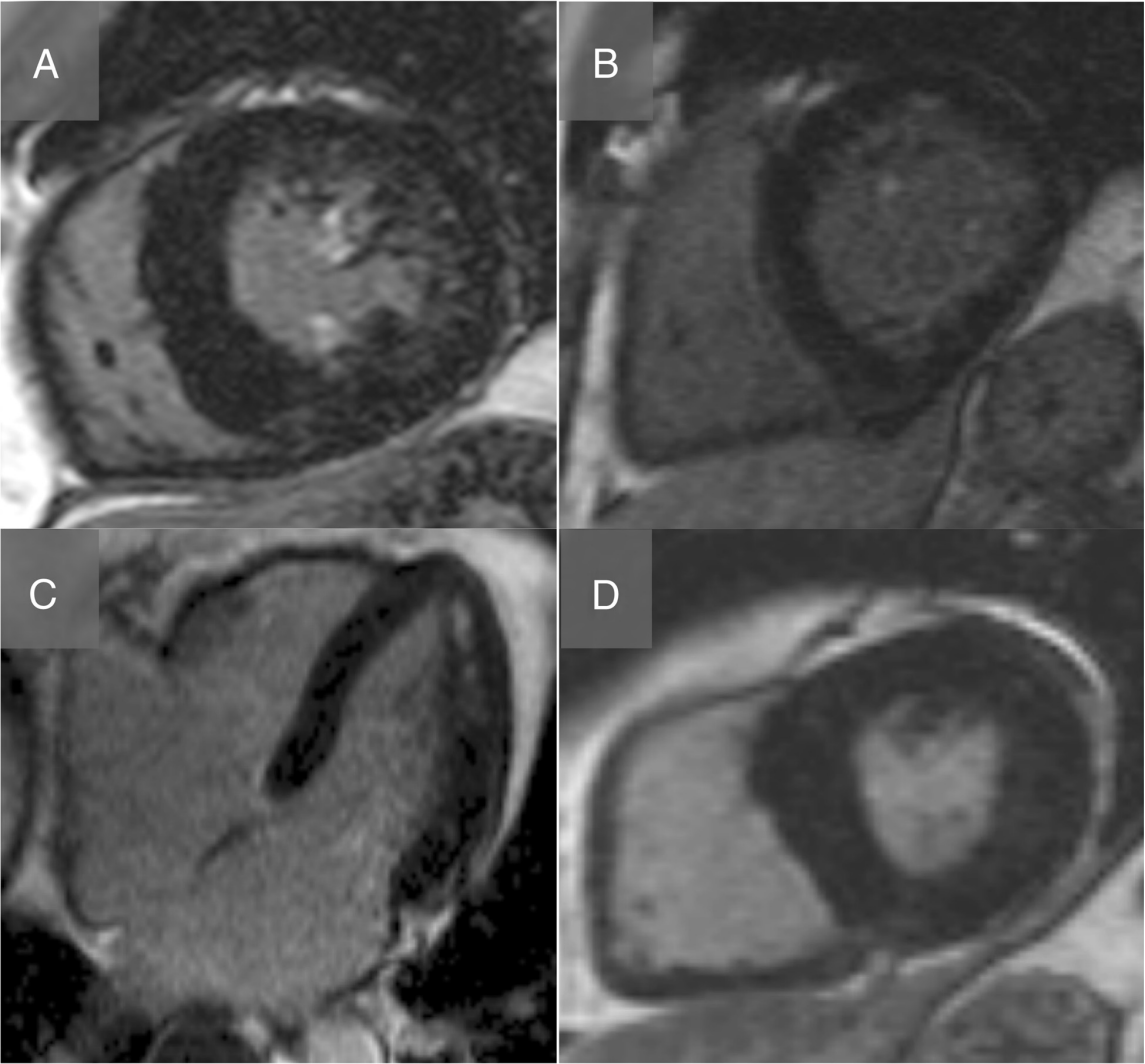
Non-ischaemic, non-HCM pattern of late gadolinium enhancement. Standard late gadolinium enhancement assessment using a fast low angle single shot inversion recovery sequence revealed non-ischaemic, non-HCM pattern of focal fibrosis in 13 % of the hypertensive cohort, with LGE in the papillary muscles (A + B) mid-wall (B + C) and right ventricular insertion points (**d**).

On LGE imaging, and after exclusion of the six patients with infarct and HCM LGE patterns (Fig. 2), 11 of the remaining 40 hypertensive subjects (28 %) had non-specific LGE either at the right ventricular insertion point, papillary muscle or patchy mid-wall enhancement (Fig. 3). These LGE patterns were seen more often with increasing degrees of LVH, evidence of cardiac remodelling (higher LV mass index, mass volume ratio, wall thickness) and increased afterload (higher systolic and diastolic pressure, and also augmentation index), but not with age, sex or ethnicity. Healthy controls did not have LGE.

### T1 mapping and ECV quantification

Native myocardial T1, equilibrium-contrast T1 and ECV values are shown in Table 5. Native myocardial T1 was similar in patients and controls (955 ± 30 ms versus 965 ± 38 ms; *p = 0.16*), however native myocardial T1 times were longer in patients with LVH than in ones without LVH (997 ± 27 ms vs. 948 ± 31 ms; *p < 0.001).* Equilibrium-contrast myocardial T1 was shorter in patients than controls (578 ± 37 ms vs. 618 ± 33 ms; *p < 0.0001)*. The difference was attributable to higher weight (86.6 ± 15.7 kg vs. 75.9 ± 13.8 kg, *p < 0.01*), which resulted in higher absolute total contrast dose, and to worse eGFR (81 ± 21 ml/min/m^2^ vs. 91 ± 17 ml/min/m^2^, p < 0.05), which also shortened equilibrium-contrast blood T1 (478 ± 56 ms vs. 517 ± 48 ms; *p < 0.001*).

**Table 5:** T1 Mapping Parameters

	Healthy Volunteers	Hypertensive	
	(n = 50)	(n = 40)	*p value*
Pre-contrast/Native			
T1 Blood (ms)	1568 ± 71	1581 ± 72	*0.4*
T1 Myocardium (ms)	955 ± 30	965 ± 38	*0.16*
Post-contrast at Equilibrium			
T1 Blood (ms)	517 ± 48	478 ± 56	*<0.001*
T1 Myocardium (ms)	618 ± 33	578 ± 37	*<0.001*
ECV by ShMOLLI (%)	26.1 ± 2.4	27.1 ± 2.7	*0.06*

The difference in ECV between the hypertensives and controls did not reach significance (27.1 ± 2.7 % vs. 26.1 ± 2.4 %, *p = 0.06*). Hypertensive patients with LVH had higher ECV than the ones without LVH (28.8 ± 2.8 % vs. 26.2 ± 2.2 %, *p < 0.01*).

The relationship between T1 mapping and clinical parameters are summarized in Table 4:

Longer **native myocardial T1** was associated with the presence of LVH (β = 44.3, *p < 0.0001*) and LVH measures: higher maximal wall thickness (β = 2.89, *p < 0.01*) and LV mass index (β = 0.4, *p < 0.01).* Native myocardial T1 was also prolonged with higher E/e’ ratio in the lateral wall (β = 3.2, *p < 0.01)*, higher central aortic pressure (β = 0.7, *p < 0.01*) and higher ECG voltage sum by Cornell (β =1.13, *p = 0.01*). The associations with indexed left atrial area (β = 3.12, *p = 0.06*), E/A ratio (β = −0.15, *p = 0.99*) and E/e’ septum (β = 1.81, *p = 0.14*) were not significant.

**Equilibrium-contrast myocardial T1** was shorter in older subjects (β = −0.09 per year, *p < 0.001),* in Afro-Caribbean/blacks *(*β = −37.5, *p < 0.05*) and subjects with LVH *(*β =2.65, *p < 0.001).* Equilibrium-contrast myocardial T1 shortened by 5 ms for each millimetre increase in maximal wall thickness (*p < 0.001*), by 7.6 ms for 10 mmHg higher systolic blood pressure (*p < 0.001*) and by 11.5 ms for each 10 mmHg higher diastolic blood pressure (*p < 0.01*). It was also shorter with worsening eGFR *(*β = 0.88, *p < 0.0001)* and strongly associated with equilibrium-contrast blood T1 time *(*β = 0.66, *p < 0.0001),* however after adjustment, only the equilibrium-contrast blood and myocardium T1 times remained their association *(*β = 0.6, *p < 0.0001*). There was neither an association between equilibrium-contrast myocardial T1 and left atrial area (β = −0.59, *p = 0.77*) nor parameters of diastolic dysfunction.

**ECV** was higher in women *(*β = 1.27, *p < 0.05)* and Afro-Caribbean/blacks *(*β = 4.1, *p < 0.0001).* Taller subjects had lower ECV *(*β = −0.05 per cm, *p < 0.05).* One % increase in haematocrit was associated with 0.23 % reduction in ECV (*p < 0.01*). Similarly to equilibrium-contrast myocardial T1, ECV increased with LV hypertrophy: maximal wall thickness *(*β = 0.18 per mm, *p < 0.05*), LV mass index *(*β = 0.02, *p < 0.05*) and with Cornell’s index *(*β = 0.12, *p < 0.05*). ECV also increased by 0.34 % for each 1 cm/m^2^ increase in LAA (but there was no significant association with parameters of diastolic dysfunction). ECV was independent of eGFR and blood pressure values. After adjustment only Afro-Caribbean race *(*β = 2.94, *p < 0.01*), height *(*β = −0.06, *p < 0.05*) and native T1 time *(*β = 0.04, *p < 0.0001*) were associated with ECV.

### Functional status and biomarkers

ECV also predicted **functional status** was predicted by ECV, with a one per cent in ECV shortening the distance on the 6-MWT by 14 meters (*p < 0.05*). Native and equilibrium T1 times were not associated with worsening functional status.

***NT-pro-BNP*** was only available for the hypertensive cohort and ranged between 4 and 100 (median 8 pmol/L). NT-pro-BNP was higher in hypertensives with LVH than without LVH (26.9 ± 16.4 vs 12.0 ± 19.6 pmol/L, *p < 0.001*). Log-transformed NT-pro-BNP correlated very weakly with markers of DMF (native myocardial T1, β = 0.005, *p < 0.01*; equilibrium-contrast T1 β = −0.004, *p < 0.05*; ECV β = 0.05, *p < 0.05*).

***Collagen biomarkers*** were available for ten healthy volunteers and 31 patients. PICP (43.0 ± 3.9 vs 54.3 ± 2.3ug/L, *p = 0.02*) and PIIINP (3.7 ± 0.1 vs 4.5 ± 0.2ug/L, *p < 0.01*) were higher in patients than in the healthy controls. There was no significant difference between LVH + ve and LVH-ve hypertensive patients. There was no association between collagen biomarker levels and myocardial T1 mapping parameters.

## Discussion

This study has shown that DMF assessed by T1 mapping increases in hypertensive patients, but that the changes were small, and occurred only in those with LVH, in keeping with prior findings using other modalities [26].

This study was of well-controlled isolated hypertension. Conventional CMR with LGE frequently adds value by identifying other pathologies – here that value was unexpectedly high – 13 % of patients had new diagnoses of hypertrophic cardiomyopathy or silent infarction. Although echocardiography is the first imaging modality for the assessment of LVH due to greater availability and smaller cost, CMR is the gold standard for LV volume and function assessment and has the advantage of allowing tissue characterisation with LGE and T1 mapping.

After exclusion of these other pathologies, 11 of the remaining 40 hypertensive subjects (28 %) had non-specific patchy LGE either at the right ventricular insertion point, in the papillary muscles or LV mid-wall enhancement. Although there is no data on the predictive value of non-ischaemic LGE in isolated arterial hypertension, it tracked increasing degree of LV hypertrophy and LV afterload. Furthermore, non-ischaemic LGE in the prototypical LV afterload disease of aortic stenosis is predictive of mortality [27].

Hypertensive patients had lower equilibrium-contrast myocardial T1 and a trend to higher ECV. Native myocardial T1 and ECV values (but not post-contrast T1) were higher in hypertensives with than without LVH. Native T1 is a composite signal of the intra- and extra-cellular myocardial compartments, whereas after administration of contrast (equilibrium-contrast T1 and ECV) the signal from the extra-cellular space dominates. The lower equilibrium-contrast T1 values in hypertensives were attributable to increased weight (increased contrast bolus) and worse renal function. The results suggest that hypertensive patients have increased myocardial fibrosis (both focal and diffuse), but that really this is triggered with the onset of LVH rather than earlier in the pathogenesis. There was some signal from those without LVH – but rather than being an early sign, this could be residual from fibrosis prior to LVH regression. In contrast to recent findings by Kuruvilla et al. [28], who found reduced peak systolic circumferential strain in LVH + ve hypertensive patients compared to LVH-ve and controls, we did not find a significant difference, which may be due aggressive anti-hypertensive treatment with associated anti-fibrotic effects (70 % of LVH + ve patients were on an ACE-inhibitor/angiotensin II or aldosterone receptor antagonist).

Ultimately, the size of the study, severity of the disease and level of control of hypertension (65 % of our subjects had no LVH) rendered this study under-powered to further elucidate all the questions; specifically, there was a lack of significant and consistent association across parameters of T1 mapping and diastolic dysfunction as well as strain. Diffuse fibrosis appears not to be a major early player in treated hypertensive heart disease prior to the onset of LVH. A larger prospective study would be necessary to scrutinise changes prior to and post therapy.

The T1 mapping field is rapidly advancing. The signal is very large in amyloid, increased in iron load, Anderson-Fabry disease, myocarditis and focal fibrosis (e.g. infarction), but smaller in diffuse fibrosis. This study suggests current technology is hitting limits for subtle changes, such as might be seen in well-controlled hypertension without LVH. Several positive studies have been published [29–31], but highlighting technological limits is as important and this effectively negative study has taken longer to get submitted than mainly positive studies, a common bias in new research fields.

### Study limitations and perspective

Patients were recruited from a specialist hypertension clinic and therefore had very well-controlled disease with documented good adherence to medication. A significant number of patients were on anti-hypertensive medications, which are attributed to have anti-fibrotic effects, which may have resulted in a reduction of LVH and ECV compared to untreated subjects, however the sample size was too small to explore this. We used good technology – ShMOLLI T1 mapping with equilibrium imaging. But the use of the equilibrium infusion technique, which requires re-positioning of the patient and does not allow co-registration of pre and post contrast maps, is not necessary for this disease. We now know the equilibrium is un-necessary for low ECV increases [32, 33]: recent work by our group and others has shown that the bolus-only technique has good agreement with histological fibrosis, with a small degree of ECV overestimation in the high ECV range [32–34]. Whether the latest techniques with new adiabatic pulses, sampling schemes, motion correction, new reconstructions (e.g. T1* for blood), ECV mapping and new methodologies to minimise partial voluming errors will make a difference is unknown currently [35].

## Conclusion

In well-controlled hypertensive patients, conventional CMR discovered significant underlying diseases (chronic infarction and HCM). T1 mapping based assessment suggested small increases in DMF, occurring only in those patients with LVH. This study highlights that interstitial changes in early hypertension (pre-LVH) are small and not detectable by current iterations of T1 mapping technique.

## ENDNOTE - Biomarker description

*Carboxy-terminal propeptide of procollagen type I (PICP):* Levels were measured in EDTA plasma by sandwich enzyme linked immunosorbent assay (ELISA) KIT manufactured by USCN Life Science Inc, Democratic Republic of China). The minimal detectable level of 26.6 pg/mL.

*Amino-terminal peptide of type III procollagen (PIIINP):* Levels were measured in EDTA plasma using a competitive radioimmunoassay (RIA) assay manufactured by Orion Diagnostica, Finland. The minimal detectable level was 0.3ug/L.

## Abbreviations

ABPM: Ambulatory blood pressure measurement
CMR: Cardiovascular magnetic resonance
DMF: Diffuse myocardial fibrosis
ECG: Electrocardiogram
ECV: Extracellular volume
EDV: End-diastolic volume
EF: Ejection fraction
ESV: End-systolic volume
HCM: Hypertrophic cardiomyopathy
LGE: Late gadolinium enhancement
LAA: Left atrial area
LV: Left ventricle
LVH: Left ventricular hypertrophy
MVR: Mass to volume ratio
NHS: National Health Service
PICP: Procollagen type I carboxy-terminal propeptide
PIIINP: Procollagen type III n-terminal propeptide
PWV: Pulse wave velocity
ROI: Region of interest
ShMOLLI: Shortened modified look-locker inversion recovery technique
SV: Stroke volume
6MWT: 6 minutes walk test
